# Healthcare-associated infections in a neonatal ICU before and during COVID-19: preliminary results: Mariateresa Ceparano

**DOI:** 10.1093/eurpub/ckac131.202

**Published:** 2022-10-25

**Authors:** M Ceparano, A Sciurti, C Isonne, MR De Blasiis, V Imeshtari, V Cammalleri, A Romano, RK Bellomo, C Marzuillo, P Villari

**Affiliations:** Public Health and Infectious Diseases, Sapienza, Rome, Italy

## Abstract

**Background:**

Healthcare-associated infections (HAIs) are a frequent complication in neonatal intensive care units (NICUs). Hospital policies caused by COVID-19 pandemic may have played a role in HAIs development. The aim of this study was to describe and characterize over time the occurrence of HAIs in a NICU at the Policlinico Umberto I in Rome both before and during the COVID-19 pandemic.

**Methods:**

All infants of all birth weight (BW) classes with >2 days in the NICU, admitted from January 2018 to December 2021, were included. To assess the effect of the pandemic, we compared surveillance data from 2018-2019 with those from 2020-2021. Infections were defined using standard Centers for Disease Control and Prevention definitions.

**Results:**

We included 513 infants, 274 admitted in 2018-2019 and 239 between 2020-2021. NICU stay in days was similar in the two periods (14.4 and 15.3 respectively) but the number of patients who died in 2018-2019 (N = 13) was almost double that of 2020-2021 (N = 7). A total of 27 infections were recorded in the post-pandemic period compared to 9 recorded in the previous period, mainly central line-associated bloodstream infections (CLABSI) (7% vs 3.0%, p = 0.043), followed by ventilator-associated pneumonias (VAP) (3.0% vs 0.4%, p = 0.019). The incidence density of device-associated infections was higher in patients with lower BW class in both periods analyzed. Different microorganisms were isolated: in 2018-2019 K. pneumoniae (33.3%) and Serratia marcescens (33.3%) were the most found, while S. aureus (29.0%) and Staphylococci coagulase negative (51.6%) were predominant in the following years.

**Conclusions:**

Results indicate that patient management may have influenced the occurrence of HAIs during the pandemic. This reinforces the importance of the HAI surveillance protocol in the NICU, which monitors microbiologic isolates and medical device use for all classes of infants with BW.

**Key messages:**

• The Covid-19 pandemic has resulted in an increase in healthcare-associated infections occurrence in our neonatal intensive care unit.

• Monitoring device-associated infections in all BW classes of infants is critical to prevent nosocomial infections.

